# Femoral Adamantinoma: A Rare Lesion in an Elderly Patient in a Rare Location

**DOI:** 10.4274/balkanmedj.2016.1534

**Published:** 2017-03-28

**Authors:** Bahattin Kerem Aydın, Zeeshan Khan, Turgay Er, Serdar Uğraş

**Affiliations:** 1 Department of Orthopaedics, Selçuk University School of Medicine, Konya, Turkey; 2 Clinic of Oncology, Royal Orthopaedic Hospital, Birmingham, United Kingdom; 3 Department of Orthopaedics and Traumatology, Rehman Medical Institute, Peshawar, Pakistan; 4 Department of Orthopaedics, ISOM Medical Center, İstanbul, Turkey; 5 Department of Pathology, Selçuk University School of Medicine, Konya, Turkey

## To The Editor,

Adamantinoma is a low-grade, extremely rare primary malignant bone tumour suggested to be of epithelial lineage. These tumours commonly present at between 10 and 50 years of age and have a propensity for presentation in the tibia (approximately 90% cases), although they have been reported in various other bones (1).

**Figure 1:**
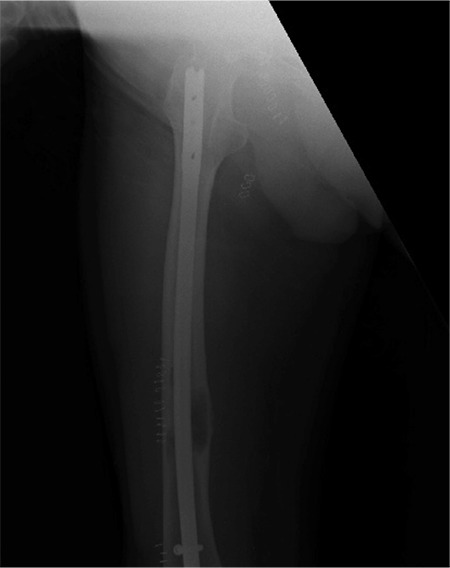
Locked intramedullary nail in situ performed for lytic lesion at primary hospital.

A 76-year-old male patient was referred to our unit from another hospital for further management of persistent pain in the femur following locked intramedullary nailing for a lytic lesion in his right femur (Figure 1). This patient presented to the primary hospital with a 2-year history of thigh and knee pain. He was managed by a general orthopaedic surgeon, who after history and examination performed a biopsy and locked intramedullary nailing simultaneously, assuming this to be a metastatic deposit, considering his age and a impending fracture.

Histology was reported at the primary hospital to be consistent with metastatic adenocarcinoma from prostate as the patient was also having treatment for prostate carcinoma. He persisted with pain in the thigh and knee 6 months following surgical management of his prostate cancer and femoral lesion and was referred to us for opinion and management.

As the patient had persistent pain, we performed a ‘metastatectomy and cementation’ of this lesion and also arranged for adjuvant radiotherapy, assuming that the whole femur was contaminated. Samples from surgery performed were reported as adamanatinoma of the bone and this was later found to be consistent with histology slides obtained from the primary hospital.

The patient was informed of the misdiagnosis. The patient refused any further surgery (hip disarticulation or endoprosthetic replacement of the whole femur) based on symptom improvement, ability to mobilise independently without any walking aids and his age. To our knowledge, this is the oldest case in English literature of a patient with adamantinoma of the femur. He was followed up in our clinic for 36 months with serial radiographs and was free of disease at the last follow-up. Written informed consent was obtained from the patient.

Some case reports of femoral adamantinoma exist, one of which describes a 60-year-old lady (2) and in another paper a 72-year-old patient (3). Surgery is the mainstay treatment and involves en bloc resection with wide margins and reconstruction (4). There is no role for curettage of the lesion, as this leads to marginal excision and inadequate margins. In our case, the patient was mismanaged because of the wrong reporting of histology. Biopsy was not conducted prior to the ‘metastatectomy and cementation’ as adamantinoma was not at all suspected in a patient of this age.

This case report describes the difficulty in diagnosing this rare lesion, which is particularly rare in elderly patients. It reminds us of the fact that one should never drift from the basic oncological principles in the management of a suspicious lesion. All these patients should be treated at a specialist unit with multidisciplinary expertise to avoid surprises. All previous investigations should be reviewed prior to planning and offering any further treatment.
